# Chemsex and Psychosis: A Systematic Review

**DOI:** 10.3390/bs12120516

**Published:** 2022-12-15

**Authors:** Lucía Moreno-Gámez, Daniel Hernández-Huerta, Guillermo Lahera

**Affiliations:** 1 Department of Medicine and Medical Specialties, University of Alcalá, 28801 Alcalá de Henares, Spain; 2 Department of Psychiatry, University Hospital Ramón y Cajal, 28034 Madrid, Spain; 3 Department of Psychiatry, University Hospital Príncipe de Asturias, 28805 Alcalá de Henares, Spain; 4 Mental Health Networking Biomedical Research Centre (CIBERSAM), 28029 Madrid, Spain; 5 Ramón y Cajal Institute of Sanitary Research (IRYCIS), 28034 Madrid, Spain

**Keywords:** chemsex, slamsex, men who have sex with men, psychosis, mental health

## Abstract

Chemsex is presented as a major challenge in public health, with numerous physical and mental consequences. The general objective of this review was to analyze the relationship between the practice of chemsex and the development of psychosis. A mixed systematic review model was chosen. PubMed, PsycINFO, and Web of Science databases were searched following a predetermined search strategy. The studies were selected, and their information was extracted following a systematic method. A total of 10 articles were included. Psychotic symptoms ranged from 6.7% to 37.2%, being one of the most frequent psychiatric diagnoses. Slamsex, polydrug use and smoked methamphetamine posed up to a 3-fold increased risk of psychosis within this practice. The risk factors found were foreign or ethnic minority status, location in large cities, stress and anxiety, trauma, loneliness, sexually transmitted infections (STIs), hepatitis, and previous psychotic history. In conclusion, chemsex is associated with psychosis development; we found numerous converging risk factors and a clear mediating role of drugs. It is important, in approaching the prevention and treatment of this addiction, to take into account motivations and psychosocial circumstances.

## 1. Introduction

Chemsex, defined at the European Chemsex Forum in Paris 2019 [1] as “the use of specific drugs in sexual contexts by gay men, bisexuals, men who have sex with men (MSM) and trans* people”, is a major international public health challenge [2]. Although there are reports of chemsex use in groups such as trans people, the profile of chemsex users tends to be that of a single gay man, aged 25–45, highly educated, and employed [3].

Mephedrone, γ-hydroxybutyric acid/γ-butyrolactone (GHB/GBL) and methamphetamine are very characteristic drugs in chemsex sessions [3]. However, polydrug use is common [4]. The key element of these sessions is their duration, which can last for days, and in which it is common to have sex with multiple partners and to engage in risky behavior such as intravenous drug use (known as slamsex) [5].

The growth of this phenomenon has been closely linked to the proliferation of geosocial networking dating apps, which facilitate both contact with potential sexual partners and the acquisition of substances [6,7]. Following Stuart (2019), engagement in chemsex is not necessarily problematic nor addictive, being important in promoting a person’s agency and autonomy to make choices based on their own assessment of the consequences [8]. Chemsex can enhance men’s capability to have the sex they want by increasing libido, sexual arousal and performance, pleasure, facilitation of sexual relations, euphoria, empathy, socialization, self-esteem, confidence, and disinhibition [4,9].

However, the practice of chemsex has been associated with legal problems; family, social, and emotional deterioration; medical risks such as overdose, interactions with other treatments and non-adherence; as well as increased incidence of infections such as HCV, HIV, syphilis, and gonorrhea, among other complications [10]. A progressive increase in the number of psychiatric consultations and admissions related to chemsex practice has also been described, with substance abuse disorders, depression, and anxiety as the most prevalent diagnoses [11].

Several risk factors associated in the scientific literature with the development of psychotic disorders are found in the practice of chemsex: drug use [4], sexually transmitted infections [12,13], stressful events and circumstances [14,15], experiences of trauma and post-traumatic stress [16,17,18,19], and loneliness [20], among others. Nevertheless, the relationship between psychosis and chemsex has been poorly investigated. To the best of our knowledge, no systematic review on the matter has been carried out to date.

### Aims

The present research aims to analyze the relationship between the practice of chemsex and the development of psychotic symptoms and disorders. The secondary objectives are to study the incidence of psychotic symptoms and disorders in people who practice chemsex and the risk factors associated with their development.

## 2. Materials and Methods

The guidelines for the publication of systematic reviews of the Preferred Reporting Items for Systematic Reviews (PRISMA), 2020, [21] were followed for this review.

### 2.1. Information Sources and Search Strategy

Following Vassar et al. (2017), to minimize the chance of including a biased sample of studies in systematic reviews, we searched more than two databases: PubMed, PsycINFO, and Web of Science (WOS) [22]. Medical Subject Headings (MeSH) in PubMed and thesauri in PsycINFO were used. PubMed was searched under “All fields”, PsycINFO under “Any field”, and WOS under “Topic”, selecting in the latter under “All databases” the WOS Core Collection and SciELO Citation Index. The last search date was 25 July 2022. The bibliographic references of the selected articles were also checked for eligibility.

In formulating the research question, a modified form of the PICO (population, intervention, comparison intervention, outcome) strategy was applied [23] (Table 1).

**Table 1:** PICO strategy.

Population	Risk Factor	Result
MSM, trans	Chemsex	Psychosis

MSM: men who have sex with men.

Within these generic boxes in the table, different terms were combined, collected for maximum transparency and reproducibility in Appendix A, which describe the different adaptations of the search strategy in each database used.

To define the population, the definition of the European Chemsex Forum in Paris 2019 [1] was used as a reference, including gay men, bisexual men, other men who have sex with men, and trans people. As “chemsex” is a very recent concept, an equivalent “Sex AND Drugs” structure box was created in order to cover the practices of this phenomenon and increase the exhaustiveness of the search, resulting in the following terminology search box scheme: MSM/trans AND (Chemsex OR (Sex AND Drugs)) AND Psychosis. Although the drugs most associated with chemsex are methamphetamine, mephedrone, and GHB/GBL, there is use of other substances, so drugs that are also frequently used in sessions, such as cocaine, were included in the “Drugs” box. In order not to lose items that might contain information of interest to the study, the terms “Mental health” and “Mental disorders” were included in the “Psychosis” box. The word “psychosis” refers here to a range of conditions that affect the mind, in which there has been some loss of touch with reality, including hallucinations, delusions, and thought disorganization. In this review, we have included studies about substance-induced psychotic disorder, schizophrenia spectrum, and other psychotic disorders following DSM-5 [24], as well as psychotic symptoms without meeting diagnostic criteria for a mental disorder.

The research question was therefore formulated as follows: what is the relationship between the practice of chemsex among MSM/trans people and the development of psychosis?

### 2.2. Study Selection Process

**Figure 1:**
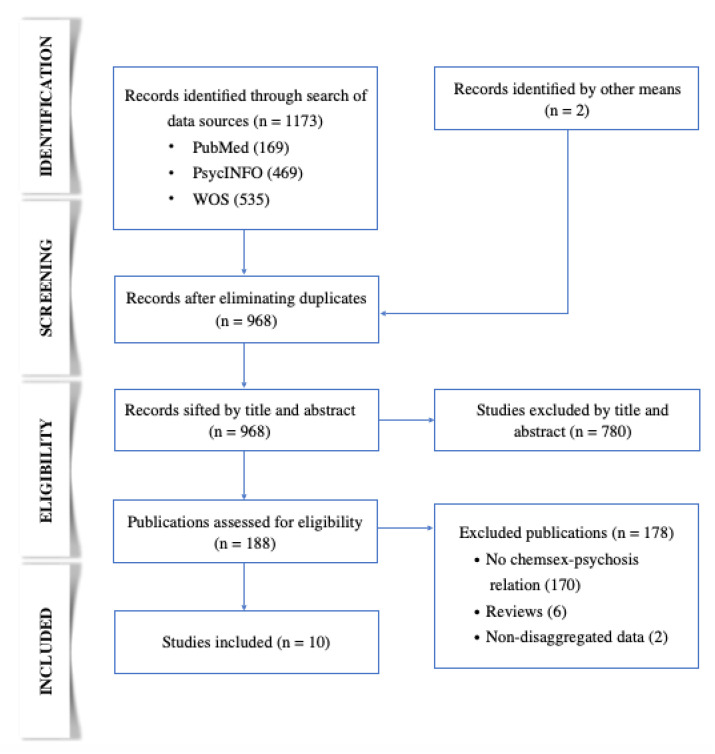
PRISMA diagram illustrating the article selection process.

In a first identification phase, the results of the databases were unified in Zotero, and duplicate records were removed. Subsequently, in the screening phase, the first author selected articles that potentially matched the inclusion criteria based on title and abstract. In the eligibility phase, after a thorough reading of the articles resulting from the previous phase, the first author made the selections, consulting with the second author in case of doubt. Any disagreement was resolved after a reasoned discussion. In the inclusion phase, the selected articles were defined and prepared for data extraction. The selection process is reflected in Figure 1.

### 2.3. Eligibility Criteria

Given the complexity of the chemsex phenomenon and seeking a broad view of the problem in question, this systematic review was defined as mixed, taking advantage of the complementarity of quantitative, qualitative, and mixed studies. The articles had to include a quantitative and/or qualitative relationship between the practice of chemsex and/or sexualized drug use among MSM and trans people and the development of psychotic symptoms and disorders. No restriction was made by language, country of origin of the study, or date of publication. Articles that addressed drug use outside a chemsex context or by population groups other than those covered by this review were excluded, as well as those that did not establish any relationship with the development of psychosis.

### 2.4. Data Extraction Process

The data from the included studies were extracted and unified in a structured table in which the following variables were collected: author, type of study, country, population, and drugs (Table 2). All authors reviewed the entire process.

### 2.5. Risk of Bias Assessment of Individual Studies

The quality of the included studies was assessed according to the Critical Appraisal Skills Programme (CASP) [25,26]. This tool is considered to be a user-friendly choice for researchers and is endorsed by Cochrane and the World Health Organization [27]. The questions in the checklist’s are divided in four parts: Are the results of the study valid? What are the results? Will the results help locally? Is the study methodologically sound?

### 2.6. Synthesis Methods

A narrative approach was used to synthesize the data obtained, following the guidance of Popay et al. (2006) [28].

**Table 2:** Studies selected characteristics (I).

Author and Year of Publication	Type of Study	Country	Population	Drugs
Bourne et al. (2015) [29]	Qualitative	United Kingdom	MSM N = 30 Mean age = 36 years	Mephedrone 90% (66.7% with GHB/GBL) and methamphetamine 33% (normally with GHB/GBL)
Dolengevich et al. (2016) [30]	Case Report	Spain	MSM aged 25	Mephedrone
Gavín et al. (2021) [31]	Retrospective longitudinal descriptive	Spain	MSM from an addiction unit N = 53 Mean age = 37.7 years	As main drug: methamphetamine 81.1%, GHB 9.4%, cocaine 3.8%, mephedrone 1.9%, speed 1.9%, and ketamine 1.9% Other substances: alcohol, cannabis, Viagra, poppers, and benzodiazepines
Dolengevich et al. (2020) [32]	Cross-sectional descriptive	Spain	MSM N = 138 Mean age = 37.46 years	From the 62 referred to psychiatry: mephedrone 64.3%, poppers 54.8%, GHB 48.3%, erectile dysfunction drugs 35.4%, methamphetamine 32.2%, cocaine 19.3%, and ketamine 4.8%
Ballesteros et al. (2017) [33]	Cross-sectional descriptive	Spain	MSM with mephedrone use disorder N = 15 Median age = 40 years	Mephedrone 100%, cocaine 66.7%, GHB 66.7%, methamphetamine 60%, ketamine 23%, and other substances (including drugs for erectile dysfunction) 25%
Batisse et al. (2016) [34]	Cross-sectional	France	MSM N = 51 Mean age = 40 years	Slamsex: mephedrone 51%, cocaine 3.9% and MDMA 2%. Non-slamsex: cocaine 33%, GHB/GBL 13%, poppers 11%, cannabis 11%, methamphetamine 8%, ketamine 8%, MDMA 6%, benzodiazepines 6%, LSD 2%, ethyl chloride 2%, and drugs for erectile dysfunction
Schreck et al. (2020) [35]	Cross-sectional	France	MSM who practiced slamsex N = 34 Median age = 38 years	Cathinones 100%, GBL 32.4%, poppers 14.8%, GHB 14.7%, cocaine 11.8%, cannabis 8.8%, methamphetamine 5.9%, MDMA 2.9%, ketamine 2.9%, and alcohol 2.9%
Bohn et al. (2020) [36]	Cross-sectional	Germany	MSM and trans (N = 3) N = 280 Mean age = 40.22 years	Poppers 87.9%, erectile dysfunction drugs 76.1%, GHB/GBL 73.6%, alcohol 72.1%, MDMA 59.6%, amphetamines 57.5%, ketamine 55.7%, methamphetamine 46.4%, cannabis 53.2%, cocaine 43.6%, mephedrone 35%, opioid analgesics 5.4%, and heroin 1.1%
Hibbert et al. (2021) [37]	Qualitative	United Kingdom	MSM N = 13 Median age = 34 years	Mephedrone 38%, poppers 38%, methamphetamine 31%, GHB/GBL 31%, cocaine 23%, and LSD 8%
Dolengevich et al. (2019) [38]	Cross-sectional descriptive	Spain	HIV+ MSM N = 216 Median age = 38 years	Cocaine 79.1%, poppers 78.7%, GHB 71.7%, cathinones 69.4%, MDMA 48.6%, ketamine 36.1%, and methamphetamine 29.6%

MSM: men who have sex with men; GHB: γ-hydroxybutyric acid; GBL: γ-butyrolactone; MDMA:3,4-methylenedioxymethamphetamine; LSD: lysergic acid diethylamide; HIV: human immunodeficiency virus.

## 3. Results

A total of ten studies that met the inclusion criteria were selected [29,30,31,32,33,34,35,36,37,38]. The selection process is reflected in Figure 1. In nine of the studies, the population consisted of MSM [29,30,31,32,33,34,35,37,38], and, in the tenth, of MSM and trans men [36]. Some studies had certain population particularities: sample from an addiction unit [31], patients with mephedrone use disorder [33], MSM who practiced slamsex [35], and HIV-positive MSM [36]. The latter study—the only study that included trans people—established comparative groups between those men who engaged in chemsex with and without slamsex, as well as those MSM and trans people that practiced chemsex with those that did not [36].

### 3.1. Sociodemographic Profile

Table 2 and Table 3 show sociodemographic characteristics of the populations of the included studies. The population samples had a mean age between 36–40.2 years old [29,31,32,34,36] and a median age between 34–40 years old [33,35,37,38]. Subjects ranged in age from 21–66 years old [29,33,34,35,37].

Of the ten studies, only the study by Bohn et al. (2020) [36] included a trans population: three trans men in the chemsex group (1.1%) and five in the non-chemsex group (2.9%). Of the samples, 17.6–69.8% belonged to a minority population subgroup or were foreign to the study site [31,36,38], 62–85% were under active employment [33,34,35,36,37], 70.8–87.2% had salaries > 1000 euros per month [36,38], 60–77.7% had higher education [33,36,38], and 42–62% reported having a stable partner [35,36,37,38].

In the study by Dolengevich et al. (2019) [38], no sociodemographic differences were observed between those who practiced chemsex without slamsex and those who practiced slamsex except for the variable “stable partner relationship”, with 45.6% in the chemsex without slamsex group and 26.5% in the slamsex group.

**Table 3:** Sociodemographic characteristics of the populations of the included studies.

Study	Foreign/Ethnic Minority Status	Under Employment	Salary >1000 €/month	Higher Education	Stable Relationship *
Bourne et al. (2015) [29]	46.7%	-	-	-	-
Gavín et al. (2021) [31]	69.8%	-	-	-	-
Dolengevich et al. (2020) [30]	-	-	-	-	-
Ballesteros et al. (2016) [33]	-	73.0%	-	60.0%	-
Batisse et al. (2016) [34]	-	85.0%	-	-	-
Schreck et al. (2020) [35]	-	62.0%	-	-	62.0%
Bohn et al. (2020) [36]	17.6%	77.8%	87.2%	77.7%	57.4%
Hibbert et al. (2021) [37]	23.0%	77.0%	-	-	46.0%
Dolengevich et al. (2019) [38]	28.7%	-	70.0%	63.9%	42.0%

-: no value available. * A sentimental relationship of a couple beyond the exclusively sexual plane, prolonged in time and with joint future plans.

### 3.2. Psychotic Symptomatology and Disorders

Table 4 collects and groups under their corresponding spectrum, psychotic symptomatology and disorders, and those other diagnoses that were described in at least two studies. The percentage of psychotic symptomatology and disorders was 6.7–37.2% [31,32,33,34,36,37,38]. Diagnoses were made by psychiatric assessment in five of the studies [30,31,32,33,35], by self-report in four studies [29,36,37,38], and in one study using both methods [34].

**Table 4:** Psychiatric symptomatology/disorders over population sample totals.

Study	Psychiatric Pathology	Psychosis	Substance Abuse/ Dependence	Anxiety	Depression	Suicidal Ideation	Suicide Attempt	ADHD
Bourne et al. (2015) [29]	16.7% ^a^	-	-	-	-	-	-	-
Gavín et al. (2021) [31]	72.1% ^b^	37.2% *	100.0% ^d^	4.7% *	20.9% *	-	-	-
Dolengevich et al. (2020) [32]	46.3%	10.1%	46.3%	3.5%	13.7%	-	-	3.1%
Ballesteros et al. (2016) [33]	60.0% ^b^	6.7%	100.0%^d^	6.7%	13.3%	-	13.3%	5.7%
Batisse et al. (2016) [34]	50.0% ^c^	31.4% *	31.4%	-	-	-	-	-
Schreck et al. (2020) [35]	26.0%	-	88.2%	8.8% *	-	2.9%	-	-
Bohn et al. (2020) [36]	-	13.2% *	-	8.3%	11.9%	12.7%	9.6%	-
Hibbert et al. (2021) [37]	-	7.7% *	-	-	-	-	-	-
Dolengevich et al. (2019) [38]	-	15.3% *	19.0%	26.9%	33.3%	15.3%	13.8%	-

-: no value available. *: Symptomatology. ^a^ Including psychosis, depression, and anxiety without quantitative values for each one. ^b^ Concomitant with substance abuse/dependence present in the entire sample. ^c^ Excluding substance abuse/dependence. ^d^ Substance abuse/dependence is one of the inclusion characteristics of the sample.

Bourne et al. (2015) [29] described paranoia and anxiety requiring medical intervention after intense chemsex sessions. The study by Hibbert et al. (2021) [37] included a case of psychosis with visual hallucinations in a 42-year-old patient after methamphetamine use. Gavín et al. (2021) [31] described delusions of self-reference (80%) and prejudice (73.3%), as well as auditory (33.3%), tactile (6.3%) and visual (6.3%) hallucinations. The evolution was of less than one month’s duration in 66.6% of those affected, and of more than six months’ duration in 20%, specifying that it was usually due to persistent use. Schreck et al. (2020) [35] also found hallucinations in the context of slamsex (2.9%) and in the days after use (8.8%). In the case report by Dolengevich et al. (2016) [30], a 25-year-old male presented with visual and kinaesthetic hallucinations, paranoid delusions, and severe anxiety after having been slamsexing with mephedrone for 3 months almost every weekend (accumulating doses of 3–4 g each), for which he required hospital admission. The study by Dolengevich et al. (2019) [38] found paranoid-type psychotic ideation in the slamsex group (29.4%) and in the chemsex group without slamsex (11%). They described that those who engaged in slamsex were up to 3 times more likely to experience psychotic symptomatology (OR = 3.37, *p* = 0.006). In the slamsex-adjusted model, anxiety (OR = 2.70, *p* = 0.042), polydrug use (OR = 2.64, *p* = 0.031) and smoked methamphetamine (OR = 3.15, *p* = 0.007) were found to be associated with the presence of psychotic symptomatology. Bohn et al. (2020) [36] described auditory hallucinations and/or paranoid symptoms, without finding a significant predictive association (Nagelkerke value R2 = 0.078) between these symptoms and previous clinical symptoms of anxiety, depression, somatisation, or post-traumatic stress, although all of them were more frequent in the chemsex group.

Regarding psychiatric history, Gavín et al. (2021) [31] found a history of unspecified psychosis in 21.4% and of substance-induced psychotic symptoms in 50%. The case report by Dolengevich et al. (2016) [30] presented a history of ADHD, antisocial behavior, and adolescent-onset substance abuse.

In relation to the approach to psychotic symptomatology, in the case of Dolengevich et al. (2016) [30], after one month of admission and treatment with paliperidone (up to 6 mg/day), zonisamide (up to 300 mg/day) for impulsive behavior and 75 mg/day of pregabalin as an anxiolytic, psychotic symptomatology completely subsided. In the study by Gavín et al. (2021) [31], 87.5% of patients were treated with second-generation antipsychotics in monotherapy, with 37.5% requiring admission for this reason at some point.

### 3.3. Trauma

Bohn et al. (2020) [36] found that the history of potentially traumatic events was 76.8% in the chemsex group. The mean number of traumatic events per person was two in the chemsex group compared to one in the non-chemsex group. They also found 11.5% of relevant PTSD (post-traumatic stress disorder) symptoms in the chemsex group.

### 3.4. Psychological and Social Motivations

The search for increased sexual ability and pleasure was one of the main motivations [29,34,35,37], with up to 92.3% of subjects reporting this motivation to start chemsex [37]. Other motives were the potential for escape (62%) and disinhibition (35%) provided by the drugs [34,35]. Similarly, the increase in security and self-esteem that drugs gave them, as well as motivations linked to the stereotype of “homosexual man with great sexual activity and multiple sexual partners”, were also present [37].

Loneliness and emptiness, with chemsex being a point of socialisation and interaction between peers, was another of the motivations given [37], as was the introduction to chemsex through sexual partners [29,37].

### 3.5. Drugs

Table 2 shows the substances consumed per study, and Table 5 shows the frequency of polydrug use and slamsex. The percentages of use in the studies in our research were:

- 35–100% mephedrone and other cathinones [29,32,34,35,36,37,38]
- 11–87.9% poppers [32,34,35,36,37]
- 5.9–60% methamphetamine [29,32,33,34,35,36,37,38]
- 11.8–79.1% cocaine [32,33,34,35,36,37,38]
- 13–73.6% GHB/GBL [32,33,34,35,36,37,38]
- 2.9–59.6% MDMA [34,35,36,38]
- 57.5% amphetamines [36]
- 2.9–55.7% ketamine [32,33,34,35,36,38]
- 8.8–53.2% cannabis [34,35,36]
- 1.1% heroin [36]
- 2–8% LSD [34,37]
- 2.9–72.1% alcohol [35,36]
- 24.9–76.1% erectile dysfunction drugs [32,36]
- 6% benzodiazepines [34]
- 5.4% opioid analgesics [36]
- 2% ethyl chloride [34]

**Table 5:** Slamsex and polydrug use.

Study	Slamsex	Polydrug Use
Bourne et al. (2015) [29]	33.3%	-
Gavín et al. (2021) [31]	-	73.6%
Dolengevich et al. (2020) [32]	40.0%	-
Ballesteros et al. (2016) [33]	53.3%	100.0%
Batisse et al. (2016) [34]	60.8%	62.0%
Schreck et al. (2020) [35]	-	85.0%
Bohn et al. (2020) [36]	30.0%	-
Dolengevich et al. (2019) [38]	15.7%	45.4%

-: no value available.

Slamming (injection) was practiced by 15.7–60.8% [29,32,33,34,36,38] and booty bumping (intra-rectal administration) by 13.3–20.4% [29,38]. The latter practice occurred in 58.8% of the slamsex group and 13.2% of the chemsex without slamsex group in the study by Dolengevich et al. (2019) [38].

Polydrug use was 45.4–100% [31,33,34,35,38], with a frequency of 82.4% in the slamsex group and 38.5% in the chemsex without slamsex group in the Dolengevich et al. (2019) study [38]. Before starting chemsex, 27–69% had a history of substance use disorder [31,34,35].

### 3.6. STIs and Infections Due to Intravenous Drug Use

Of the samples, 15.4–93% had been infected with HIV [31,32,33,34,35,36,37], 2–41% with HCV [31,33,34,35,36], 3.8–26.6% with HBV [31,33,34], 27–43% with syphilis [31,33,35], and 9.4% with gonorrhea [31].

The study by Bohn et al. (2020) [36] reported HIV prevalence of 41.2% in the chemsex group and 13.5% in the non-chemsex group but found no significant differences in the statistical analysis for HCV. Dolengevich et al. (2019) [38] found percentages in the slamsex vs. chemsex without slamsex groups for gonorrhea of 61.8% vs. 43.4%, for syphilis of 88.2% vs. 62.6%, and for HCV of 61.8% vs. 18.1%, respectively. In the case report by Dolengevich et al. (2016) [30], the patient had a history of HIV, HCV, syphilis, and genital candidiasis. In fact, he had been reinfected with HCV 3 months before admission for a psychotic condition.

Group practice predominated in the sessions, with Ballesteros et al. (2016) [33] documenting a 73.3% prevalence of the orgy form. Dolengevich et al. (2019) [38] identified that 70% had had >20 sexual partners in the last 6 months in the slamsex group and 39.6% in the chemsex without slamsex group. In the same study, prevalences of fisting of 73.5% and 38.5%, respectively, were also described for the slamsex and chemsex without slamsex groups.

Another risky behavior was the non-use of condoms, taking place on at least one occasion during chemsex sessions in 86.6% in the study by Ballesteros et al. [33]. Dolengevich et al. (2019) [38] found prevalence of condomless sex in 93.1% of the slamsex group and 48.3% of the chemsex without slamsex group. Ballesteros et al. (2016) [33] identified that 75% shared injecting equipment, while Dolengevich et al. (2019) [38] found a prevalence of 97.1% for the slamsex group.

Gavín et al. (2021) [31] reported that 96.3% of HIV patients were on antiretroviral treatment and 87.9% had an undetectable viral load. Hibbert et al. (2021) [37] found use of pre-exposure prophylaxis (PrEP) in 23% of the sample, and HIV patients considered that chemsex did not affect their adherence to treatment. In the work of Dolengevich et al. (2019) [38], more than 90% reported adherence to antiretroviral therapy, although the slamsex group had 9.1% low adherence compared to 1.9% in the chemsex group without slamsex.

### 3.7. Sexual Abuse

Bourne et al. (2015) [29] described how GHB/GBL overdoses resulted in a state of unconsciousness known as G-hole, during which 10% of men described experiencing sexual abuse. Dolengevich et al. (2019) [38] found a frequency for these losses of consciousness of 15.3%, being 29.4% among MSM who practiced slamsex and 12.6% in the chemsex group without slamsex. Bohn et al. (2020) [36] identified that 47.2% of the chemsex group had experienced situations in which they felt that their sexual partners did not respect their boundaries in the sexual scenario, compared with 26.8% in the non-chemsex group. Furthermore, 17.7% claimed to have been drugged without their consent in the chemsex group. Similarly, Hibbert et al. (2021) [37] describe an account of a patient who claimed to have been drugged in order to be abused.

### 3.8. Professional and Personal Impact

Overall, the personal and professional impact of chemsex is described as marked, ranging from the costs involved to the time spent, the fatigue it generates, and drug dependence, among others [29,36,37]. Bohn et al. (2020) [36] describe how 33.6% of those who used chemsex had missed work or had worked while still under the influence of drugs. Schreck et al. (2020) [35] found social consequences in 55% of the sample, with 44% separation and 24% job loss. Dolengevich et al. (2019) [38] identified that interference with work, social, or family life occurred in 31.5% of the sample, with a greater impact in the slamsex group (64.7%) compared to the chemsex group without slamsex (25.3%).

## 4. Discussion

The purpose of this review was to investigate the association between chemsex and psychotic symptomatology/disorders. Beyond confirming a relationship between the two, the results of the present review also allow us to describe, following a biopsychosocial perspective, a series of risk factors for psychosis associated with the chemsex phenomenon.

In our review, we found percentages of psychotic phenomenology related to chemsex ranging from 6.7% to 37.2% (Table 4), which are higher than the 5% usually observed in the general adult population [39]. We also observed that psychotic disorders were the most frequent diagnoses together with substance use disorders, depressive disorders, and anxiety disorders. Symptomatology described included delusions of paranoid [29,30,36,38], reference, and prejudice [31] types, as well as hallucinations [35] of visual [30,31,37], auditory [31,36], tactile [31], and kinesthetic [30] types. Discontinuation of consumption and the use of antipsychotics and other adjuvant drugs were effective, requiring hospital admission in 37.5% of cases [31].

A variety of drugs were used in the studies (Table 2). There are several hypotheses about the pathophysiological mechanisms by which the use of these drugs could generate psychotic symptomatology. Some hypotheses are imbalance in dopamine levels in the prefrontal cortex with cannabis, genetic variants in the dopamine transporter and in catechol-O-methyltransferase for cocaine, dysregulation in glutamate transmission pathways through the thalamocortical system with methamphetamine, action on 5-HT2A receptors with LSD, long-term neurotoxicity with MDMA, and D2 receptor affinity and inhibition of GABAergic activity in the prefrontal cortex with ketamine [40]. Among the studies providing data on the possible drug associated with the psychotic episode, we find methamphetamine [37] and mephedrone in slamsex [30]. On the other hand, the study by Dolengevich et al. (2019) [38] found a significant relationship between the use of smoked methamphetamine and the presence of psychosis (OR = 3.15, *p* = 0.007). Polydrug use (45.4–100%) and slamsex (15.7–50.8%) showed high frequency data in our review, which found a relationship between slamsex (OR = 3.37, *p* = 0.006) and polydrug use (OR = 2.64, *p* = 0.031) with the presence of psychotic symptoms [38]. In addition, polydrug use was more frequent among those who practiced slamsex [35,38], as well as booty bumping, which could have kinetics similar to those of slamming [34]. The temporal and contextual circumstances in which the psychotic symptomatology occurred, during the sessions or in the days following them, is evidence of the close relationship between drug taking and the presentation of psychotic phenomenology. The evolution was variable; 66% had a duration of less than one month, although 20% of those affected had a duration of psychotic symptomatology of more than six months, usually due to persistent drug use [31]. A frequent history of substance abuse prior to chemsex has also been observed, with figures ranging from 27–69% in our review [31,34,35].

Age is a risk factor for psychosis, with higher rates of psychotic disorders observed among young males, which is consistent with much of the population described in this review, even though the mean age in our review (36–40.2 years) (Table 2) is slightly higher than the commonly observed higher incidence for psychosis, which is around 18–24 years [41]. Another factor that has been related to the occurrence of psychotic disorders is foreign/ethnic minority status, which generates a greater number of adverse circumstances and stressors that increase the risk of psychosis [42]. In our review we observed that 17.6–69.8% of the samples (Table 3) belonged to a minority population group or were foreigners, remarkable figures to take into consideration. In relation to the results of work activity, academic studies, and economic level, these factors do not seem to be relevant in the psychosis–chemsex interaction, since most of the samples were under employment, had salaries >1000 euros per month, and had higher education (Table 3). On the other hand, the relationship between urbanicity and psychosis [43] does seem to play a relevant role in the chemsex phenomenon; most of the studies reflected in this review focused on large cities, where the chemsex phenomenon has developed the most [44].

It is difficult to differentiate the relationship between chemsex and psychosis and the well-known association between non-sexualized drug use and psychosis. Previous research has shown that men engaged in chemsex might have suffered early adverse events and might have an avoidant–insecure attachment style [45], having had previous low sexual wellbeing and mental health. Much research shows that gay and bisexual men face a multitude of distal and proximal stressors (violence, discrimination, expectations of rejection, internalized homophobia, concealment of their sexuality) across the life course, which can heighten the risk of mental health problems. This is known as the minority stress theory [46,47,48]. Moreover, it has been scientifically reported that the homosexual population is up to two times more likely to suffer from psychotic symptoms than the heterosexual population [49]. The use of chemsex has been reported as a coping mechanism for the stressors that MSM experience on a daily basis [14]. In our review we have found escape and disinhibition as reasons to practice chemsex, as well as to increase self-esteem and self-confidence [34,35,37]. However, the practice of chemsex can lead to highly stressful circumstances such as work, social, and emotional problems:

- Bohn et al. (2020) [36] identified that 33.6% of those who used chemsex had missed work or had worked while still under the influence of drugs.
- Schreck et al. (2020) [35] described that 55% of men had social problems, with 44% experiencing separation and 24% missing work.
- In the study by Dolengevich et al. (2019) [38], up to 31.5% of men reported interference with work, social, or family life, with a greater impact on the slamsex group (64.7%) compared to the chemsex group without slamsex (25.3%).

The prevalence of anxiety disorders found among the samples was 3.5–26.9% (Table 4) while that found in the general male population does not reach 3% [50]. In the study by Dolengevich et al. (2019) [38], anxiety was shown to be a predictor of the association between psychosis and chemsex (OR = 2.70, *p* = 0.042); however, the study by Bohn et al. (2020) [36] found no significant differences in anxiety, although it did show that chemsex users had higher scores on the GAD-7 scale than non-chemsex users.

Another line of research in psychosis is linked to trauma (16). In the study by Bohn et al. (2020) [36], up to 76.8% of the sample reported a traumatic history, with a mean number of traumatic events experienced per person of 2:1 between chemsex and non-chemsex users. However, no significant predictive association was found between post-traumatic stress and psychotic symptoms in the chemsex group. These findings could be related to the mediating role of chemsex in coping with trauma [17]. On the other hand, this review also reflects the situations of sexual abuse that can occur in chemsex sessions, especially in situations of overdose, a factor that has been related to the development of psychosis through the trauma generated [19].

Recently, a polygenic association between isolation and subjective loneliness and psychosis has been demonstrated [51]. Along these lines, the results found in the present review show a high percentage of men who lived alone (53.33%) [33], and loneliness and the search for socialization as reasons for attending chemsex sessions [37]; these factors could play a role in the development of psychotic phenomenology.

In relation to STIs and infections due to intravenous drug use, we found high figures in our review, especially for HIV. The risk behaviors associated with chemsex and reflected in our review, such as having multiple sexual partners, sex without condoms, fisting, and sharing injecting equipment, among others, justify the infection figures. In the study by Hibbert et al. (2021) [37], patients reported adequate adherence to PrEP, although this measure only reduces the risk of HIV infection and not against other STIs. It is also noted that slamsex practice conferred a higher risk of infections than those who practiced chemsex without slamsex [38]. At the neuropsychiatric level, some of these infections are associated with the development of psychosis; for example, opportunistic infections of the central nervous system, brain lymphomas, and encephalitis can occur in HIV and lead to secondary psychosis [52]. However, the studies in our review reported high adherence to antiretroviral therapy and undetectable viral load, although these results were lower among those who practiced slamsex [38]. Psychotic symptoms can also be found as a manifestation of neurosyphilis [8] or as a consequence of hepatitis treatment [53].

**Figure 2:**
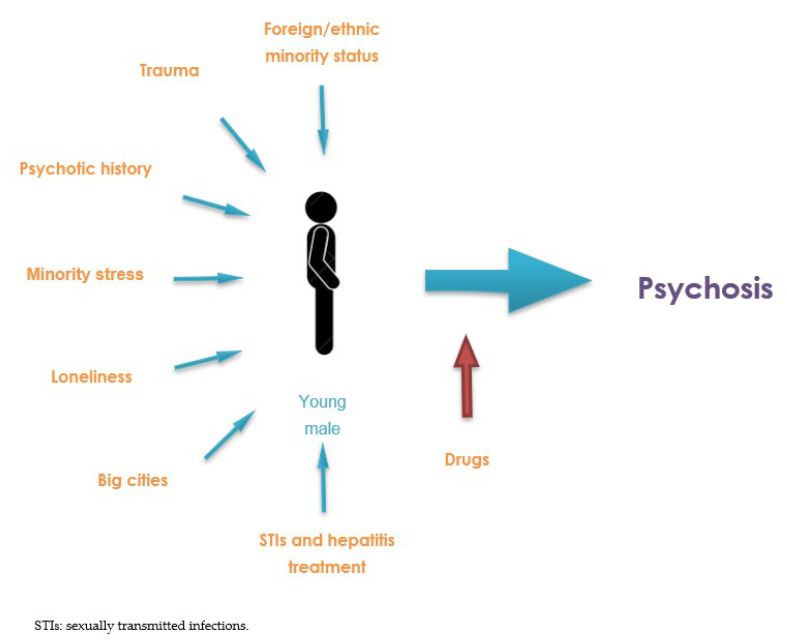
Diagram of the relationship between chemsex and psychosis.

Taking all of the above into consideration, Figure 2 provides a diagram showing the factors that could favor the development of psychosis associated with the practice of chemsex.

Some of these risk factors have a cultural and social basis, which opens the door to social changes with a potential preventive effect. For example, belonging to an ethnic minority seems to be an individual risk factor for chemsex and psychosis, but the combined effect of homonegativity and racism can critically contribute to this relation; therefore, societal and psychotherapeutic interventions for enhancing psychological well-being among gay and bisexual men should focus on building feelings of identity resilience [46]. Potential preventive interventions include a person-centered therapeutic approach that can enable the individual to focus on positive aspects of their lives that generate feelings of self-esteem, self-efficacy, continuity, and positive distinctiveness. Prevention and awareness should be promoted without leading to greater stigma. At a clinical level, it also seems important to address abstinence among those who present a related psychotic episode, given the high rate of continued use. Chemsex is a modifiable risk factor for psychosis (and many other mental and physical pathologies) and we must understand the motivations and set of psychosocial circumstances that lead our patients to practice it, approaching via active listening and looking for a reduction and alternative projection of internal discomfort.

### 4.1. Limitations and Strengths

The number of studies that analyzed psychotic symptoms and disorders related to chemsex use was small. Of the included studies, six collected psychotic symptomatology in the form of self-reports [29,36,37,38] so biases such as recall and the non-medical judgement of participants may have interfered. Moreover, in four of the studies, the samples were ≤30 subjects (Table 2), including one case report, which limits the generalizability of the results. An added limitation was the heterogeneity in collecting and reflecting the findings in the studies, as well as the presence of four articles that sampled more specific subpopulations than the one in this review, which made it difficult to unify and synthesize the information. As previously mentioned, only one study included a trans population [36]; furthermore, the fact that it did so by self-identification with male sex left out trans women, a part of the group in which a large amount of sexualized drug use is being found [54]. However, as we explained, the role of this minority group has yet to be studied, with MSM being the clear demographic that defines chemsex. Moreover, only one study included a comparative non-chemsex group [36], and another established comparative slamsex and non-slamsex chemsex groups [38], leading to less specificity in our research. Finally, the use of a mixed form of systematic review allowed us to combine quantitative and qualitative contributions, which added to the integration of biological, psychological, and social perspectives carried out in this work and allows for a more complete approach to the phenomenon.

### 4.2. Future Lines of Research

Future lines of research should better define the role of the different risk factors for psychosis within chemsex in order to understand how, beyond the action of drugs, the phenomenon with all its integrated aspects would place chemsex as a risk factor for the development of psychotic symptoms and disorders. Future research should also investigate in greater depth what motivations these subjects have for engaging in chemsex despite knowing physical and psychological consequences, as well as what preventive interventions could be effective in this field.

## 5. Conclusions

Our research concludes that there is an association between chemsex use and the risk of developing psychosis (including psychotic symptoms and psychotic disorders), and our findings suggest that this symptomatology may be more prevalent than has been suggested [11]. Similarly, there are a number of risk factors that may contribute to this association: there is a clear mediating role for drugs, with slamsex use, smoked methamphetamine, and polydrug use being associated with an increased likelihood of psychosis. Moreover, numerous additional risk factors show high prevalence and converge in chemsex as a “perfect storm”: being a young male migrant, living in big cities, stress and anxiety, trauma, loneliness, STIs, treatment for contracted hepatitis, and a history of psychotic disorders (Figure 2).

The present research should be taken into account when including psychosis among the diagnoses and complications to be considered when conducting chemsex-related investigations. Future research should elucidate and clarify the risk of developing psychosis associated with chemsex, as well as the interactions between the various risk factors present in chemsex.

## Data Availability

All data is provided in this article or in the Appendix A.

## Appendix A

- PubMed:

((((((((((((((“Sexual and Gender Minorities”[Mesh]) OR (“Homosexuality, Male”[Mesh])) OR (“Bisexuality”[Mesh])) OR (“Transsexualism”[Mesh])) OR (“Transgender Persons”[Mesh])) OR (“Gay”)) OR (Homosexual*)) OR (Bisexual*)) OR (“Men who have sex with men”)) OR (“MSM”)) OR (Transexual*)) OR (Transsexual*)) OR (Transgender*)) AND (((((((“Sexual Behavior”[Mesh]) OR (“Sexual behavio*”)) OR (“Sexual health”)) OR (“Sexual risk”)) OR (“Unsafe sex”)) AND (((((((((((((((((((((“Illicit Drugs”[Mesh]) OR (“Amphetamines”[Mesh])) OR (“Sodium Oxybate”[Mesh])) OR (“4-Butyrolactone”[Mesh])) OR (“Ketamine”[Mesh])) OR (“Cocaine”[Mesh])) OR (“Nitrites”[Mesh])) OR (“Sildenafil Citrate”[Mesh])) OR (“Cannabis”[Mesh])) OR (MDMA)) OR (Ecstasy)) OR (Mephedrone)) OR (Methamphetamine)) OR (Amphetamine)) OR (GHB)) OR (GBL)) OR (Ketamine)) OR (Cocaine)) OR (Poppers)) OR (Viagra)) OR (Cannabis))) OR ((((((Chemsex) OR (Slamsex)) OR (“Sexualized drug*”)) OR (“Sexualized drug*”)) OR (“Party and play”)) OR (“Intensive sex partying”)))) AND ((((((((((((((((“Mental Health”[Mesh]) OR (“Mental Disorders”[Mesh:NoExp])) OR (“Schizophrenia Spectrum and Other Psychotic Disorders”[Mesh])) OR (“Delusions”[Mesh])) OR (“Hallucinations”[Mesh])) OR (“Catatonia”[Mesh])) OR (“Mental health”)) OR (“Mental disorder*”)) OR (Psychosis)) OR (Psychotic)) OR (Schizo*)) OR (Paranoi*)) OR (Delusion*)) OR (Hallucination*)) OR (“Negative symptom*”)) OR (Catatoni*))

- PsycINFO:

(MAINSUBJECT.EXACT(“Mental Health”) OR MAINSUBJECT.EXACT(“Mental Disorders”) OR MAINSUBJECT.EXACT(“Catatonia”) OR MAINSUBJECT.EXACT(“Delusions”) OR MAINSUBJECT.EXACT(“Hallucinations”) OR MAINSUBJECT.EXACT.EXPLODE(“Psychosis”) OR “Mental health” OR “Mental disorder*” OR Psychosis OR Psychotic OR Schizo* OR Paranoi* OR Delusion* OR Hallucination* OR “Negative symptom*” OR Catatoni*) AND (((MAINSUBJECT.EXACT.EXPLODE(“Psychosexual Behavior”) OR “Sexual behavio*” OR “Sexual health” OR “Sexual risk” OR “Unsafe sex”) AND (MAINSUBJECT.EXACT.EXPLODE(“Cannabis”) OR MAINSUBJECT.EXACT.EXPLODE(“Cocaine”) OR MAINSUBJECT.EXACT(“Sildenafil”) OR MAINSUBJECT.EXACT(“Gamma Hydroxybutyrate”) OR MAINSUBJECT.EXACT.EXPLODE(“Amphetamine”) OR MAINSUBJECT.EXACT.EXPLODE(“Drug Usage”) OR MAINSUBJECT.EXACT(“Ketamine”) OR MDMA OR Ecstasy OR Mephedrone OR Methamphetamine OR Amphetamine OR GHB OR GBL OR Ketamine OR Cocaine OR Poppers OR Viagra OR Cannabis)) OR (Chemsex OR Slamsex OR “Sexuali?ed drug*” OR “Party and play” OR “Intensive sex partying”)) AND (MAINSUBJECT.EXACT(“Male Homosexuality”) OR MAINSUBJECT.EXACT(“Bisexuality”) OR MAINSUBJECT.EXACT(“Transsexualism”) OR MAINSUBJECT.EXACT(“Transgender”) OR MAINSUBJECT.EXACT(“Sexual Minority Groups”) OR Gay? OR Homosexual* OR Bisexual* OR “Men who have sex with men” OR MSM OR Trans?exual* OR Transgender*)

- WOS:

#8 AND #34 AND #45
8: #1 OR #2 OR #3 OR #4 OR #5 OR #6 OR #7
  1: TS = (Homosexual*)
  2: TS = (Gay$)
  3: TS = (“Men who have sex with men”)
  4: TS = (MSM)
  5: TS = (Bisexual*)
  6: TS = (Trans$exual*)
  7: TS = (Transgender*)
34: #27 OR #33
  27: #13 AND #26
  13: #9 OR #10 OR #11 OR #12
  9:TS = (“Sexual behavio*”)
  10: TS = (“Sexual health”)
  11: TS = (“Sexual risk”)
  12: TS = (“Unsafe sex”)
  26: #14 OR #15 OR #16 OR #17 OR #18 OR #19 OR #20 OR #21 OR #22 OR #23 OR #24 OR #25
  14: TS = (“Illicit drug*”)
  15: TS = (*amphetamine)
  16: TS = (MDMA)
  17: TS = (Ecstasy)
  18: TS = (Mephedrone)
  19: TS = (GHB)
  20: TS = (GBL)
  21: TS = (Ketamine)
  22: TS = (Cocaine)
  23: TS = (Poppers)24: TS = (Viagra)
  25: TS = (Cannabis)
  33: #28 OR #29 OR #30 OR #31 OR #32
  28: TS = (Chemsex)
  29: TS = (Slamsex)
  30: TS = (“Sexuali?ed drug*”)
  31: TS = (“Party and play”)
  32: TS = (“Intensive sex partying”)
45: #35 OR #36 OR #37 OR #38 OR #39 OR #40 OR #41 OR #42 OR #43 OR #44
  35: TS = (“Mental health”)
  36: TS = (“Mental disorder*”)
  37: TS = (Psychosis)
  38: TS = (Psychotic)
  39: TS = (Schizo*)
  40: TS = (Paranoi*)
  41: TS = (Delusion*)
  42: TS = (Hallucination*)
  43: TS = (“Negative symptom*”)
  44: TS = (Catatoni*)
